# Video Image Enhancement and Machine Learning Pipeline for Underwater Animal Detection and Classification at Cabled Observatories

**DOI:** 10.3390/s20030726

**Published:** 2020-01-28

**Authors:** Vanesa Lopez-Vazquez, Jose Manuel Lopez-Guede, Simone Marini, Emanuela Fanelli, Espen Johnsen, Jacopo Aguzzi

**Affiliations:** 1 DS Labs, R+D+I unit of Deusto Sistemas S.A., 01015 Vitoria-Gasteiz, Spain; 2 University of the Basque Country (UPV/EHU), Nieves Cano, 12, 01006 Vitoria-Gasteiz, Spain; 3 Department of System Engineering and Automation Control, Faculty of Engineering of Vitoria-Gasteiz, University of the Basque Country (UPV/EHU), Nieves Cano, 12, 01006 Vitoria-Gasteiz, Spain; jm.lopez@ehu.es; 4 Institute of Marine Sciences, National Research Council of Italy (CNR), 19032 La Spezia, Italy; simone.marini@sp.ismar.cnr.it; 5 Stazione Zoologica Anton Dohrn (SZN), 80122 Naples, Italy; e.fanelli@univpm.it (E.F.); jaguzzi@icm.csic.es (J.A.); 6 Department of Life and Environmental Sciences, Polytechnic University of Marche, Via Brecce Bianche, 60131 Ancona, Italy; 7 Institute of Marine Research, P.O. Box 1870, 5817 Bergen, Norway; espen.johnsen@hi.no; 8 Instituto de Ciencias del Mar (ICM) of the Consejo Superior de Investigaciones Científicas (CSIC), 08003 Barcelona, Spain

**Keywords:** cabled observatories, artificial intelligence, deep learning, machine learning, deep-sea fauna

## Abstract

An understanding of marine ecosystems and their biodiversity is relevant to sustainable use of the goods and services they offer. Since marine areas host complex ecosystems, it is important to develop spatially widespread monitoring networks capable of providing large amounts of multiparametric information, encompassing both biotic and abiotic variables, and describing the ecological dynamics of the observed species. In this context, imaging devices are valuable tools that complement other biological and oceanographic monitoring devices. Nevertheless, large amounts of images or movies cannot all be manually processed, and autonomous routines for recognizing the relevant content, classification, and tagging are urgently needed. In this work, we propose a pipeline for the analysis of visual data that integrates video/image annotation tools for defining, training, and validation of datasets with video/image enhancement and machine and deep learning approaches. Such a pipeline is required to achieve good performance in the recognition and classification tasks of mobile and sessile megafauna, in order to obtain integrated information on spatial distribution and temporal dynamics. A prototype implementation of the analysis pipeline is provided in the context of deep-sea videos taken by one of the fixed cameras at the LoVe Ocean Observatory network of Lofoten Islands (Norway) at 260 m depth, in the Barents Sea, which has shown good classification results on an independent test dataset with an accuracy value of 76.18% and an area under the curve (AUC) value of 87.59%.

## 1. Introduction

### 1.1. The Development of Marine Imaging

Over the past couple of decades, imaging of ocean biodiversity has experienced a spectacular increase [1], revolutionizing the monitoring of marine communities at all depths of the continental margins and the deep sea [2]. At the same time, a relevant development has taken place in robotic platforms bearing different types of imaging devices in association with a diversified set of environmental sensors [3]. Among these assets there are cabled observatories as video-functioning multiparametric platforms connected to shore by fiber optic cables [4]. These fixed platforms are being used to acquire image material from which animals of different species can be identified and then counted in a remote fashion, at a high frequency and over consecutive years [5,6,7,8,9]. Then, extracted biological time series are used to estimate how populations and species respond to the changes in environmental conditions (also concomitantly measured) [10,11,12,13,14].

### 1.2. The Human Bottleneck in Image Manual Processing

Although a large amount of scientific literature has been produced in recent years about underwater content-based image analysis [15,16,17,18,19], the processing of video data within ecological application contexts is still mostly manual and cabled observatory platforms and their networks are not yet equipped with permanent software tools for the automated recognition and classification of biological relevant image content [3,20]. In this context, cabled observatory networks such as Ocean Network Canada (ONC, www.oceannetworks.ca), European Multidisciplinary Seafloor and water-column Observatories (EMSO, http://emso.eu/), and Lofoten-Vesterålen (LoVe, https://love.statoil.com/) among others, are missing the opportunity to increase their societal impact by enforcing service-oriented image acquisition and automatically processing target species of commercial relevance. 

At the same time, the development of artificial intelligence (AI) oriented to image treatment for animal counting and classification requires the development of analysis tools for ecological annotation, as well as semantic infrastructures for combining datasets and recognition and classification algorithms [21]. To do this, it is necessary to create relevant and accessible ecological repositories (e.g., Fish4Knowledge, http://groups.inf.ed.ac.uk/f4k/ and SeaCLEF, https://www.imageclef.org/) and, in them, to define effective ground-truth datasets for an effective classification [6]. 

### 1.3. Objectives and Findings

The cabled Lofoten-Vesterålen (LoVe) observatory network is located in the Norwegian deep sea, in the Norwegian continental slope of Lofoten Islands, in the Barents sea at 260 m depth, an area hosting one of the highest abundance cold-water coral (CWC) reefs in the world [22,23,24]. CWCs host a rich associated fauna [25], especially fish, with several species of high commercial value for the local fishery, such as the rockfish *Sebastes* spp. [26]. Presently, the monitoring of its local population and other species in the surrounding community has not yet been undertaken, which is relevant to the production of some ancillary data for fishery-independent and ecosystem-based management models (e.g., how fish respond to other species or oceanographic variations and how this is reflected in commercial availability).

Within the envisaged development of the LoVe observatory network, aiming to establish a science-based infrastructure for continuous online monitoring of the ocean interior including benthic, pelagic, and the demersal habitats, we propose a user-friendly integrated library of tools (specifically developed for that cabled observatory network), aimed at the following: (i) The generation of ground-truth datasets through semi-automatic image annotation, (ii) the training of supervised underwater image classifiers based on ground-truth datasets, and (iii) the automated classification of underwater images acquired by cameras installed on fixed and mobile platforms. These tools support the video/image analysis of the LoVe still imaging outputs dedicated to the tracking and classification of different species of the local deep-sea community. 

In order to explore and maintain the wide biodiversity and life of underwater ecosystems, monitoring and subsequent analysis of the information collected is necessary. Due to the numerous underwater images, as well as videos collected at sea, manual analysis becomes a long and tedious task, therefore, this study proposes a pipeline to solve this task automatically.

The objective of this study is to introduce a pipeline for underwater animal detection and classification, which includes image enhancing, image segmentation, and manual annotation (to define training and validation datasets), and automated content recognition and classification steps. This pipeline has demonstrated good results in the classification of animals of the Norwegian deep sea, reaching an accuracy value of 76.18% and an area under the curve (AUC) value of 87.59%.

The paper is organized as follows: Section 2 presents the dataset used in this work, and describes the processing pipeline and the experimental setup, within the chosen evaluation metrics; Section 3 shows the obtained results; while Section 4 introduces the discussion about the preliminary results; and finally, Section 5 presents our conclusions. 

## 2. Materials and Methods

### 2.1. The Cabled Observatory Network Area

**Figure 1:**
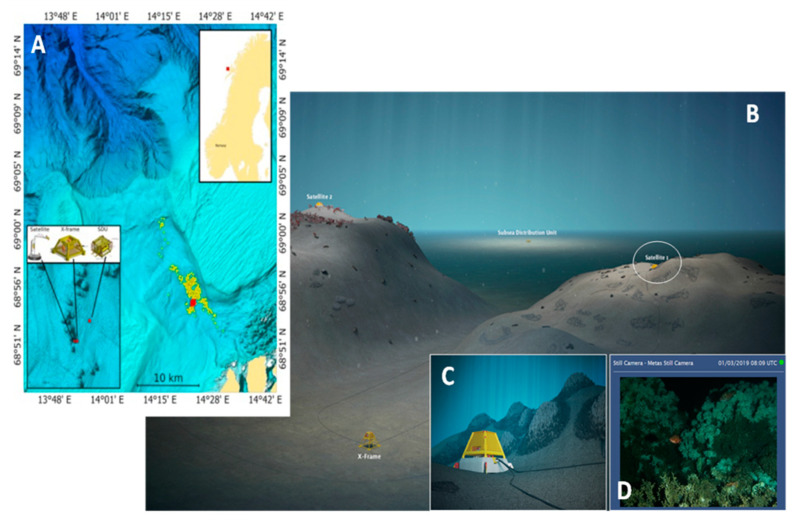
Overview of the study area where the Lofoten-Vesterålen (LoVe) observatory is located: (**A**) Bathymetric map of the canyon area showing (in red) the observatory area and (in yellow) relevant *Desmophyllum pertusum* reef mounds around it (adapted from [24]), (**B**) three-dimensional (3D) detailed representation of the area showing (encircled in white) the video node providing the footage used to train AI procedures, (**C**) enlarged view of the areas surrounding the node where *D. pertusum* reefs are schematized, and finally (**D**) the field of view as it appears in the analyzed footages (B, C, and D) taken from the observatory site at https://love.statoil.com/.

The LoVe observatory is located 20 km of the Lofoten Islands (Norway) in the Hola trough (Figure 1) and was deployed in 2013. This glacially deepened trough is 180 to 260 m deep and incises the continental shelf in a northwest to southeast direction from the continental slope to the coast. The location of the observatory (∼260 m deep) is enclosed by two 100 m deep banks, Vesterålsgrunnen in the northeast and Eggagrunnen in the southwest. The trough has a diverse topography with sand wave fields of up to 7 m high, 10 to 35 m high ridges, and approximately 20 m high CWC mounds [27]. The CWC mounds are predominantly found in the southeastern part of the trough at a depth of ∼260 m just south of the Vesterålsgrunnen bank, being mostly constituted by CWC *Desmophyllum pertusum* [28].

The following three platforms compose the data collection system of this area: The X-Frame, which measures water current and biomass in water (with an echosounder); Satellite 1, which collects multiple types of data, such as photos, sound, chlorophyll, turbidity, pressure, temperature, conductivity, etc.; and Satellite 2, which only collects photos. The images used in this paper were acquired with Satellite 1 (see also Section 2.3).

### 2.2. The Target Group of Species 

The area around the LoVe observatory is rich in biodiversity, and the following species were identified according to the local fauna guides [29,30,31] (Figure 1 and Table 1): *Sebastes* sp., *Lithodes maja, Sepiolidae, Bolocera tuediae, Pandalus* sp., *Echinus esculentus*, *Brosme brosme, Cancer pagurusa,* and *Desmophyllum pertusum*. Some other species were also targeted by our automated protocol but could not be classified and are generically categorized as ”hermit” crab and ”starfish”.

**Figure 2:**
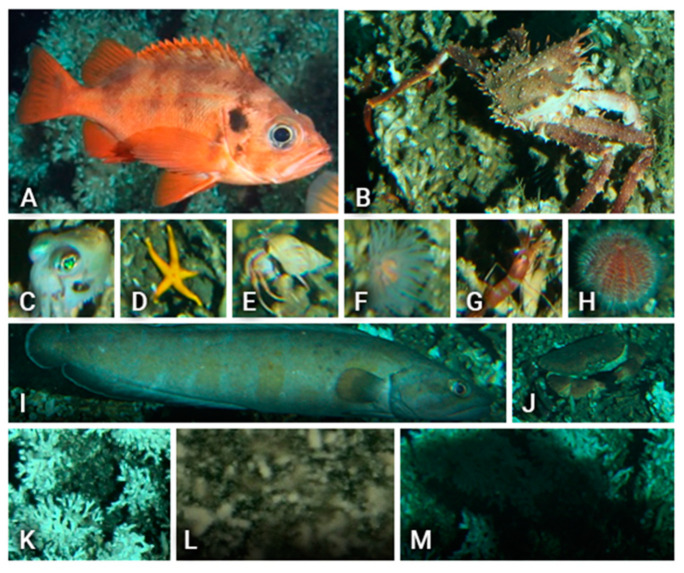
An example of video-detected species used for building the training dataset for reference at automated classification: (**A**) Rockfish (*Sebastes* sp.), (**B**) king crab (*Lithodes maja*), (**C**) squid (*Sepiolidae*), (**D**) starfish, (**E**) hermit crab, (**F**) anemone (*Bolocera tuediae*), (**G**) shrimp (Pandalus sp.), (**H**) sea urchin (*Echinus esculentus*), (**I**) eel-like fish (*Brosme brosme*), (**J**) crab (*Cancer pagurus*), (**K**) coral (*Desmophyllum pertusum*), and finally (**L**) turbidity, and (**M**) shadow.

Among these species, only *Sebastes* (Figure 2) has commercial importance. This is a genus of fish in the family Sebastidae, usually called Rockfish, encompassing 108 species, two of them (*Sebastes norvegicus* and *Sebastes mentella*) inhabiting Norwegian deep waters and presenting very similar morphological characteristics including coloring [32]. *Sebastes norvegicus* has been reported in LoVe *Desmophyllum* areas up to six times with higher density as compared with the surrounding seabed [25,29]. Accordingly, we referred to *Sebastes* sp. for all rockfish recorded at the LoVe observatory. 

Another two elements were selected due to their abundance in the footage, turbidity, and shadows. The so-called ”turbidity” class refers to the cloudiness sometimes seen in water containing sediments or phytoplankton, while the ”shadow” class corresponds to the shadows cast by some of the fish.

### 2.3. Data Collection

The images used for testing the proposed tools were extracted from time-lapse footages (image acquisition period of 60 min) generated with photos obtained by the camera from Satellite 1 (see previous Section 2.1 and Figure 1) in two time windows, the first from 4 October 2017 to 27 June 2018 and the second from 10 December 2018 to 29 June 2019. Accordingly, a total of 8818 images were available continuously over the 24 h period during 372 consecutive days. Some images were missing due to the observatory structure maintenance.

### 2.4. Image Processing Pipeline for Underwater Animal Detection And Annotation 

The images provided by LoVe observatory were acquired in an uncontrolled environment, characterized by a heterogeneous background of coral bushes, where turbidity and artificial lighting changes make it difficult to detect elements with heterogeneous shapes, colors, and sizes.

**Figure 3:**
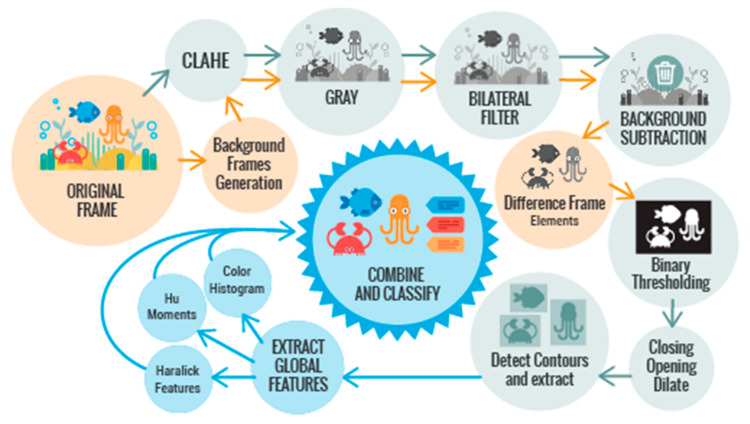
Image processing pipeline.

An image processing pipeline (Figure 3) was designed and developed based on computer vision tools for enhancing the image contrast and for segmenting relevant image subregions [19,33]. To speed up this process, the images were resized from 3456 × 5184 pixels to a quarter of their size, i.e., 964 × 1296 pixels.

First, a background image was generated for each day, that is, obtaining the average of the 24 images for each 24 h. These images were used to perform the background subtraction after applying different techniques to the images.

The contrast limited adaptative histogram equalization (CLAHE) technique [34] was applied to enhancing the image background/foreground contrast. While the traditional adaptive histogram equalization [35] is likely to amplify noise in constant or homogeneous regions, the CLAHE approach reduces this problem by limiting the contrast amplification using a filtering technique [36,37,38]. After this equalization, a bilateral filtering [39] was applied in order to discard irrelevant image information while preserving the edges of the objects that are to be extracted.

The background subtraction took place at this time. In this way, a frame was obtained with only the elements detected in the original image.

A binary thresholding value, which was chosen by testing different values, was performed to obtain the mask of the elements in the image [19,33,40,41,42,43] and different morphological transformations such as closing, opening, and dilation were applied to remove noise. 

Global features were extracted for subsequent classification, which is explained later.

Finally, the contours of the threshold image were detected in order to identify the relevant elements in the input image. The whole process was carried out with Python, OpenCV [44], and Mahotas [45].

As the size of the collected set was only a total of 1934 elements, we decided, first, to apply data augmentation techniques to 80% of the images (a total of 1547 images), which are the ones that made up the training set.

Data augmentation involves different techniques in order to generate multiple images from an original one to increase the size of the training set. Within this work, several image transformations were used such as image flipping, rotation, brightness changes, and finally zoom. After applying data augmentation techniques, the training set increased from 1547 to 39,072 images.

Because global features such as texture or color features have obtained good results in the classification task in the literature [46,47,48], we extracted and combined several global features from all images, which are summarized in Table 2.

For the classification part, several algorithms were compared with each other to clarify which one obtained a more accurate classification result. Traditional classifiers such as support vector machine (SVM), k-nearest neighbors (K-NN), or random forests (RF) have been widely used for underwater animal classification. For example, in [52], the authors made a comparison between many classical algorithms obtaining an accuracy value higher than 0.7. Another study reached 82% of correct classification rate (CCR) with a SVM [53]. In recent years, deep learning (DL) approaches [54] have gained popularity due to their advantages, as they do not need the input data to be processed and often they get better results for problems related to image quality, language, etc. [23]. Accordingly, we decided to make a comparison between both types of methods; evaluating the results and performance of four classical algorithms and two different neural networks.

SVM is a supervised learning approach that can perform both linear or nonlinear classification or regression tasks [55,56,57] and has shown good results in the classification of underwater image features [58,59].

K-NN is a fast algorithm that classifies an object by a majority vote of its *k* (a positive integer) nearest neighbors [60], being a recurrent classifier used in this domain [40,53].

Decision trees (DTs) are algorithms that perform both classification and regression tasks, in addition, they use a tree structure to make decisions [61,62]; each middle leaf (called node) of the tree represents an attribute, the branches are the decisions to be made (by rules), and each leaf of the tree that is a final node, corresponds to a result. This kind of classifier is also popular in underwater animal classification, thus, the obtained results are quite good [63,64].

RF is an ensemble of DTs [65,66]. It normally applies the technique of bootstrap (also called bagging) at training. It uses averaging of the DT results to improve the predictive accuracy and to avoid over-fitting. Although RF have not been used as much as other algorithms, they have shown their performance and results [67].

Convolutional neural networks (CNNs or ConvNets) have shown good accuracy results solving underwater classification problems [68,69,70]. Deep neural networks (DNNs) have also been used successfully in this field [71].

Different structures, training parameters, and optimizers were chosen in order to make a comparison between them and determine which of the combinations obtained the best results. This is described in the next section.

### 2.5. Experimental Setup

Two versions of SVM were selected. The first one is a linear SVM (LSVM) with C = 1, which is a parameter used to determine the margin that separates the hyperplane for classification and influences the objective function. The second one is also a LSVM but with stochastic gradient descent (SGD) [72] training, which is an iterative method commonly used for optimizing, with hinge as the loss function and elasticnet [73] as the regularization term.

Regarding the K-NN algorithm, for the selection of the *k*-value, three criteria were considered as follows:

The selected number is preferable to be odd in order to avoid confusion between two classes;

The *k* value should be accurately selected, since small values could lead to overfitting and large values would make the algorithm computationally expensive [74,75];

The approximation used to set the ***k***-value was the result of calculating the square root of the total number of samples in the training set [74], following Equation (1)

(1) $$k\;=\;\sqrt{\#\;samples\;training\;set}.$$

Using the last criteria, two K-NN classifiers were tested, one with ***k*** = 39 and the other ***k*** = 99.

As was explained in the previous section, DTs have gained popularity and two DTs were chosen. For the proposed analysis, the selected number of nodes between the root and the leaves, was 3000 and 100,000.

Regarding RFs, two different RFs were selected, each with different parameters. The first one with 75 trees, 300 nodes, and 10 features to consider when performing the splitting; the second one with 50 trees, 1000 nodes, and 50 features.

The implementation of all the classical algorithms used are within the Scikit-learn library [76] (https://scikit-learn.org).

In the case of the DL approach, we selected four CNNs and four DNNs.

Two different structures were selected for the four CNNs. The first structure (CNN-1 and CNN-3) was composed of two blocks of convolution, activation, and pooling layers, while the second one (CNN-2 and CNN-4) contained three blocks. The activation function selected was rectified linear unit (ReLU), which is a commonly used function with CNNs. The four models have fully connected layers at the end, with an activation layer bearing a softmax function, which is a categorical classifier widely used in DL architectures [68]. For training, two different optimizers were selected. For the CNN-1 and CNN-2, Adadelta [77] was used and for the second group, CNN-3 and CNN-4, RMSProp was used [78]. The training parameters, such as epochs and batch size, were established on the basis of initial tests in which it was observed that Networks 1 and 2 (which have the optimizer in common) reached high accuracy values in the early epochs, while CNN-3 and CNN-4 took longer to improve their accuracy. In this way, for CNN-1 and CNN-2 the number of epochs was 50 and the batch size was 356. For the other two networks, CNN-3 and CNN-4, the number of the epochs was 150 and the batch size was decreased to 128.

The DNNs models have a similar layer structure. Similar to the previous network groups, the first structure (corresponding to DNN-1 and DNN-3) contains an input layer followed by three dense layers, each one followed by one activation layer. The first activation layer contains a ReLU function, whereas the others have a hyperbolic tangent function (tanh). Even this function is not as common as ReLU because it can cause training difficulties, it has obtained good results with some optimizers such as SGD [79]. These layers are followed by a dropout layer to prevent overfitting [80]. The second structure (for DNN-2 and DNN-4) is basically the same as the previous one but has one layer more and the activation function for each layer is the ReLU function. This time, RMSPprop and SGD were selected as the optimizers. As DNNs can be trained faster than the CNNs, the number of epochs selected was 500 for all DNNs, while the batch size was 518 for DNN-1 and DNN-2 and 356 for DNN-3 and DNN-4. A summary of the experimental setup of the DL models is shown in Table 3.

Each one of the networks was fed with the extracted global features from each element of the training dataset. These features were stacked together in a one-dimensional (1D) array. The output of each of the networks is one of the 13 classes defined in Table 1.

The environment used for training the selected algorithms and the defined models was Google Colaboratory (also known as Colab). Colab operates currently under Ubuntu 18.04 (64 bits) and it is provided by an Intel Xeon processor and 13 GB RAM. It is also provided with a NVIDIA Tesla K80 GPU. Traditional algorithms were trained on CPU, while deep learning models were trained on GPU.

### 2.6. Metrics

On the basis of the choices made by some studies in the literature of similar scope [47,76], every classifier was validated by 10-fold cross-validation by considering that the elements of each class were distributed evenly in each one of the folds. The performance of the models was evaluated by the accuracy, loss, and area under the curve (AUC) average scores [81].

The accuracy is given by Equation (2):

(2) $$Accuracy=\frac{TP+TN}{P+N}=\frac{TP+TN}{TP+FP+TN+FN}$$

where ***TP*** is true positive, ***TN*** is true negative, ***FP*** is false positive, ***FN*** is false negative, ***P*** is real positives, and ***N*** is real negatives.

**Figure 4:**
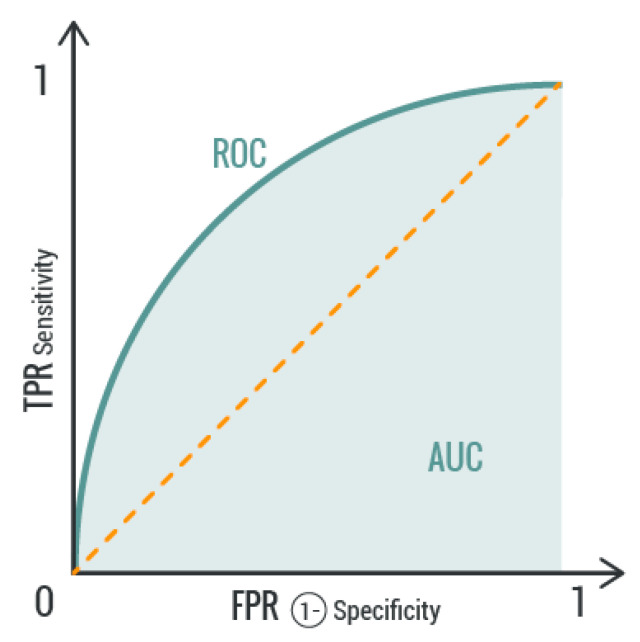
Receiver operating characteristic (ROC) curve.

The AUC measures the area underneath the receiver operating characteristic (ROC) curve, as shown in Figure 4:

The true rate positive (***TPR***) or sensitivity is given by Equation (3), while the false rate positive (***FPR***) or specificity is defined by Equation (4):

(3) $$TPR=\frac{TP}{P}=\frac{TP}{TP+FN}$$

(4) $$FPR=\frac{FP}{N}=\frac{FP}{FP+TN}$$

The accuracy and AUC values were calculated by the macro called averaging technique, which calculates metrics for each label, without considering the label imbalance.

The loss function measures the difference between the prediction value and the real class. It is a positive value that increases as the robustness of the model decreases.

## 3. Results

**Figure 5:**
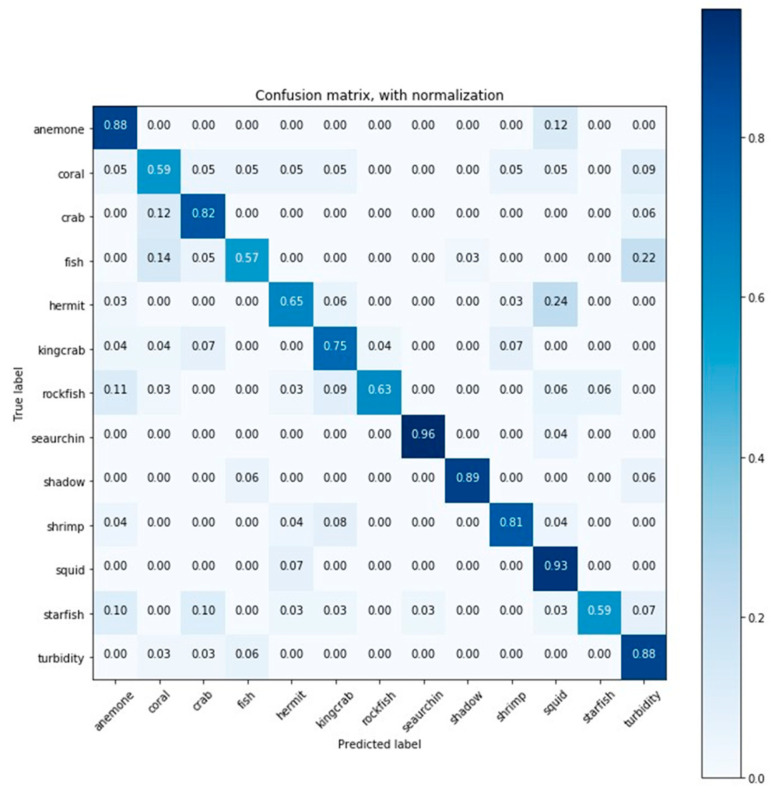
Confusion matrix for the classification results (accuracy) obtained by random forest (RF) RF-2.

**Figure 6:**
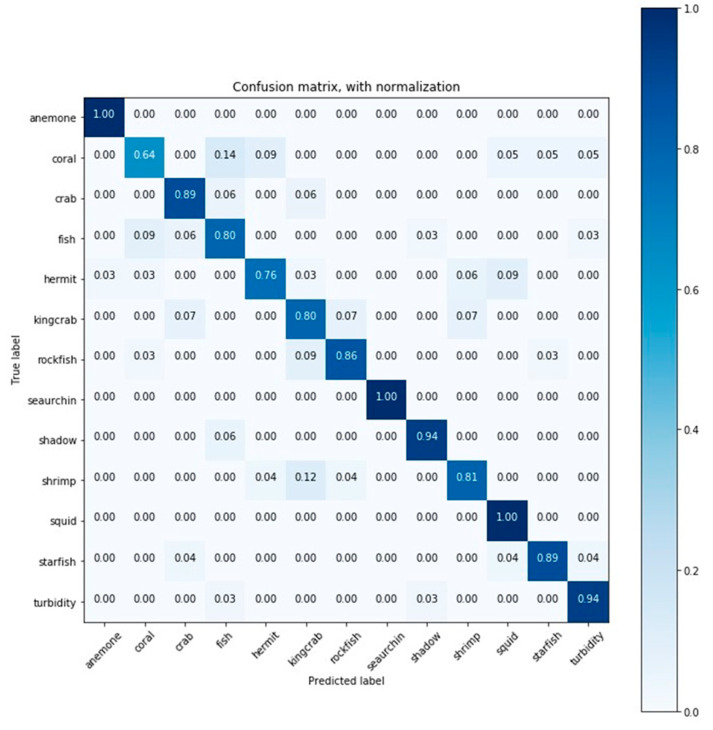
Confusion matrix for the classification results (accuracy) obtained by deep neural network (DNN) DNN-1.

Accuracy and AUC average values obtained for all classes and for each classifier were obtained performing cross-validation. The average training time is also shown in Table 4. The obtained confusion matrices of RF-2 and DNN-1 are summarized in Figure 5 and Figure 6 respectively, while the remaining detailed results are found in the supplementary material (Appendix A). 

Referring to traditional classifiers, the worst result was reached by K-NN with k = 99, as it barely reached an AUC value of 0.6390. However, the other K-NN (***k*** = 39) achieved better results, as it reached an AUC value of 0.7140. The two DTs and the RF-1 performed quite well, as they almost achieved an AUC of 70%. The linear SVM reached an AUC of 0.7392 but also had the longest training time, which was 1 minute and 11 seconds. The SVM with the SGD optimization function did not work as well as the linear SVM, as it barely reached an AUC value of 0.6887. The RF-2 gained the highest AUC value, 0.8210, using a short training time of 8 s. The accuracy values are much lower for every classifier as compared with the AUC values.

The DL approaches obtained better results than almost every other traditional classifier. The eight networks obtained AUC values from 80% to 88%. CNNs achieved an AUC values between 0.7983 and 0.8180. The four DNNs obtained results between 0.8361 and 0.8759, respectively. Similar to the case of the accuracy values obtained by traditional classifiers, the accuracy achieved by DL approaches was also lower than AUC values. However, despite being lower values, all neural networks exceeded values of more than 60%; and most of the DNNs exceeded accuracy values of 70%.

The confusion matrix of Figure 5 corresponds to the results obtained by RF-2, were the X axis shows the predicted label and the Y axis shows the true label. For some classes, such as anemone, crab, sea urchin, shadow, shrimp, squid, and turbidity worked well, as it predicted values correctly between 70% to 93%. Coral, fish and starfish classes were misclassified by 59%, 57%, and 59%. Other classes such as hermit crab, king crab, and rockfish were also misclassified, but at least 60% of the elements were correctly classified.

Figure 6 shows the confusion matrix for the classification results obtained by DNN-1, which achieved good results for almost every class. In this case, three classes (anemone, sea urchin, and squid) were classified correctly at 100%, and the worst ranked class (coral) had 64% correctly labeled.

**Figure 7:**
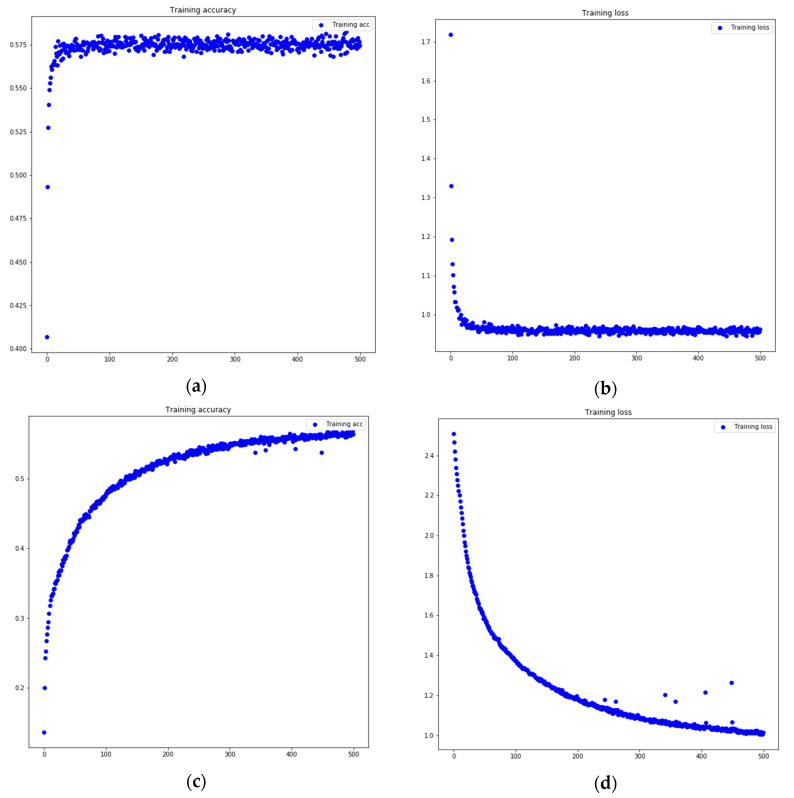
Training accuracy and loss plots of the DNNs with different structures. The X axis of all of plots shows the number of epochs, while the Y axis show the loss or accuracy value that reached the trained model. Training accuracy and loss plots of DNN-1: (**a**) Accuracy values obtained in every epoch at training time and (**b**) loss values obtained in every epoch at training time. Training accuracy and loss plots of DNN-4: (**c**) Accuracy values obtained in each epoch at training time and (**d**) loss values obtained in each epoch at training time.

The performance of the four DNNs had different accuracy and loss values during the training, as shown in Figure 7a,b. The first two, which are the ones that obtained the best results, in the first 50 epochs, had already reached an accuracy value close to the final value (just over 0.60) and at the same time, the loss value also decreased to the final minimum value reached. 

However, both DNN-3 and DNN-4 took a longer amount of epochs to reach the highest accuracy value, as well as the lowest loss value, as shown in Figure 7c,d. As it progressively reached more optimal values, it did not reach the best values until at least 450 epochs.

DNN-1 was used to extract the time series of organism abundance, that is, it was used to detect, classify, and count animals in a short period of time in order to compare that result with the ground truth. This was performed on images not used during the training and test phase, corresponding to the period from 17 November 2017 to 22 November 2017.

**Figure 8:**
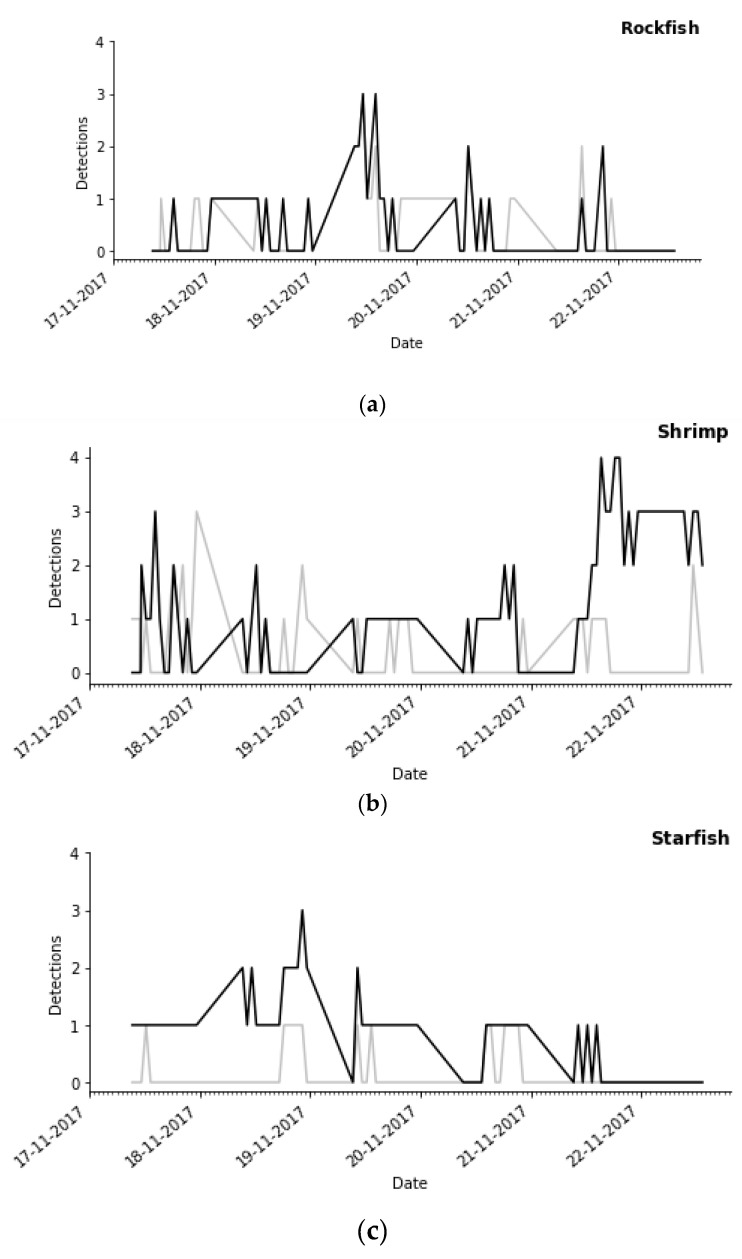
Time series of detections per day of (**a**) rockfish, (**b**) starfish, and (**c**) shrimp taxa. In the three plots, the X axis shows consecutive dates, while the Y axis shows the number of detections. The black lines correspond to the manual detection and the grey lines correspond to the estimated counts by the automatic process.

Figure 8 shows three different time series for the rockfish, shrimp, and starfish during that period of six days, which covers 80 images. The classifier detected rockfish in 27 images, whereas with the manual detection, animals were detected in 24 images, which means that there are at least three false positives. In the other time series, the difference is much higher.

## 4. Discussion

In this study, we have presented a novel pipeline that can be used in an automatic pipeline for analysis of video image with the goal of identification and classification of organisms belonging to multiple taxa. The environment is difficult due to the turbidity that can sometimes be seen in the water, which makes it hard to appreciate the species; the small size of the dataset, which limits the appearance of some of the animals; the colors of the species detected, as well as the size of some of them, which sometimes blend in with the environment. All this can sometimes lead to incorrect classifications. Despite all this, we obtained successful classification results over the thirteen different taxa that we identified.

The image preprocessing pipeline automatically extracted 28,140 elements. Among them, between 90 and 200 specimens were manually selected from the 13 different classes of organisms (Table 1).

Two different types of methods were used in this study, i.e., classical algorithms and DL techniques. In general, the training phase for a DL approach needs hundreds of thousands of examples [82,83,84] or as an alternative, it can benefit from transfer learning approaches [85,86]. On the contrary, the proposed work uses only images acquired by the LoVe observatory with the aim of using the proposed image processing tools for incrementing the training set during the operational activities of the observatory. 

Data augmentation was applied to the training dataset to obtain a richer one. The final training dataset consists of 39,072 images as follows: 2886 specimens of anemone, 3034 of coral, 3034 of crab, 3034 of fish, 3034 of hermit crab, 3034 of king crab, 3034 of rockfish, 3034 of sea urchin, 2997 of shadow, 3034 of shrimp, 2849 of squid, 3034 of starfish, and 3034 of turbidity. Similar studies also detected the advantages of DL over ML methods in marine environments [87,88,89,90].

With respect to the structures and training parameters chosen for all the networks, it can be seen that, for CNNs, the ones that obtained the best results were the CNN-2 and CNN-4, which had the same structure (the one with more layers) but different optimizers and parameters. However, in the case of DNNs, the DNN-1 and DNN-2 which share optimizer and parameters but not the same structure, obtained better results. Since the difference in results was not very large, it is necessary to perform more exhaustive experiments in order to conclude which element has the greatest influence on the results. In order to improve the pipeline and, consequently, the result, more work and in-depth study is needed.

As future work in this research line, the pipeline for the automated recognition and classification of image content introduced in this study should be permanently installed on the LoVe observatory augmented with the mobile platforms developed within the ARIM (Autonomous Robotic Sea-Floor Infrastructure for benthopelagic Monitoring) European project. The introduced pipeline could be used to notably increase the ground-truth training and validation dataset and obtain more accurate image classifiers. Within this application context, the development of neural networks could be further extended, creating models with different structures (adding and removing layers, modifying the number of units for each layer) and applying distinct parameter configuration (such as increasing or decreasing the number of epochs, batch size, and varying the chosen optimizer for training, or combining different activation functions). Other types of methods that have been proven to be successful should be considered, such as transfer learning approaches. Many studies have shown that the use of pretrained neural networks overcome results from non-pretrained neural networks [91,92]. This method is commonly used to improve the extraction of features and the classification when the training set is small [93,94], although this was not the case in this study, and it is less and less, because LoVe collects more images.

Changing the dataset would be challenging, as we could select images or videos with other characteristics, such as a moving background similar to [95], where they collected underwater sea videos using an ROV camera. Other possibilities include modifying the dataset, cropping images, or dividing fish into pieces to compare results, similar to [96].

Considering all the above, based on this work, we could make use of the transfer learning technique on a new network, and test it in other datasets.

## 5. Conclusions

The aim of this study was to design an automatic pipeline for underwater animal detection and classification, performing filtering and enhancing techniques, and using machine learning techniques. We obtained results with accuracy values of 76.18% and AUC of 87.59%, so the objective was achieved.

As can be seen in this study, our results reaffirm that unexplored underwater environments can be analyzed with the help of classic approaches and DL techniques. Moreover, DL approaches such as complex neural networks have shown that it is quite appropriate to identify and classify different elements, even if the images quality is sometimes low [74].

The improvement and enhancement of underwater images also plays an important role in detecting elements. It would be interesting to deepen in these methods, since a clear improvement of the images could reduce the later work of detection of features and obtain better classification rates. 

The use of traditional classifiers and DL techniques aimed at the detection of marine species and, consequently, their assessment, both qualitative and quantitative, of the environment corresponding to each one, demonstrates that it can be an important advance in this field.

If we contemplate the advances in the acquisition of images and other parameters in different underwater ecosystems, it is easily deduced that the amount of information provided by the different acquisition centers would be impossible to analyze if it was not through this type of automatic technique.

## Figures and Tables

**Table 1:** An example of video-detected species used for building the training dataset for reference at automated classification.

Class (alias)	Species Name	# Specimens per Species in Dataset	Image in Figure 2
Rockfish	*Sebastes* sp.	205	(A)
King crab	*Lithodes maja*	170	(B)
Squid	*Sepiolidae*	96	(C)
Starfish	*Unidentified*	169	(D)
Hermit crab	*Unidentified*	184	(E)
Anemone	*Bolocera tuediae*	98	(F)
Shrimp	*Pandalus* sp.	154	(G)
Sea urchin	*Echinus esculentus*	138	(H)
Eel like fish	*Brosme brosme*	199	(I)
Crab	*Cancer pagurus*	102	(J)
Coral	*Desmophyllum pertusum*	142	(K)
Turbidity	-	176	(L)
Shadow	-	101	(M)

**Table 2:** Extracted global features from each image.

Type	Description	Obtained Features
Hu invariant moments [49]	They are used for shape matching, as they are invariant to image transformations such as scale, translation, rotation, and reflection.	An array containing the image moments
Haralick texture features [50]	They describe an image based on texture, quantifying the gray tone intensity of pixels that are next to each other in space.	An array containing the Haralick features of the image
Color histogram [35,51]	The representation of the distribution of colors contained in an image.	An array (a flattened matrix to one dimension) containing the histogram of the image

**Table 3:** Summary of the experimental setup of the different neural networks.

**CNN-1**	**CNN-2**	**CNN-3**	**CNN-4**
Structure 1	Structure 2	Structure 1	Structure 2
Optimizer 1	Optimizer 1	Optimizer 2	Optimizer 2
Parameters 1	Parameters 1	Parameters 2	Parameters 2
**DNN-1**	**DNN-2**	**DNN-3**	**DNN-4**
Structure 1	Structure 2	Structure 1	Structure 2
Optimizer 1	Optimizer 1	Optimizer 2	Optimizer 2
Parameters 1	Parameters 1	Parameters 2	Parameters 2

**Table 4:** Accuracy and area under the curve (AUC) values with test dataset and training time obtained by different models.

Type of Approach	Classifier	Accuracy	AUC	Training Time (h:mm:ss)
**Traditional classifiers**	Linear SVM	0.5137	0.7392	0:01:11
	LSVM + SGD	0.4196	0.6887	0:00:28
	K-NN (***k*** = 39)	0.4463	0.7140	0:00:02
	K-NN (***k*** = 99)	0.3111	0.6390	0:00:02
	DT-1	0.4310	0.6975	0:00:08
	DT-2	0.4331	0.6985	0:00:08
	RF-1	0.4326	0.6987	0:00:08
	RF-2	0.6527	0.8210	0:00:08
	CNN-1	0.6191	0.7983	0:01:26
	CNN-2	0.6563	0.8180	0:01:53
**DL**	CNN-3	0.6346	0.8067	0:07:23
	CNN-4	0.6421	0.8107	0:08:18
	DNN-1	0.7618	0.8759	0:07:56
	DNN-2	0.7576	0.8730	0:08:27
	DNN-3	0.6904	0.8361	0:06:50
	DNN-4	0.7140	0.8503	0:07:16

**Figure A1:**
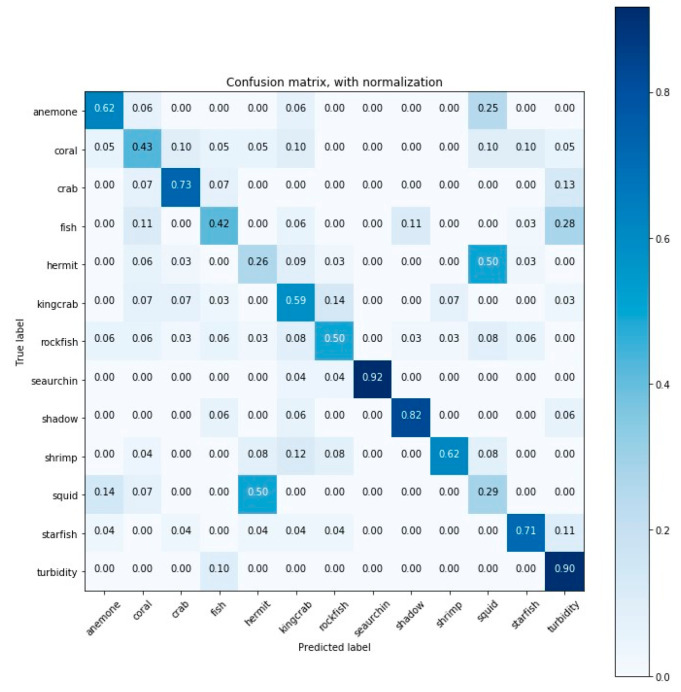
Confusion matrix for the classification results (accuracy) obtained by linear support vector machine (SVM).

**Figure A2:**
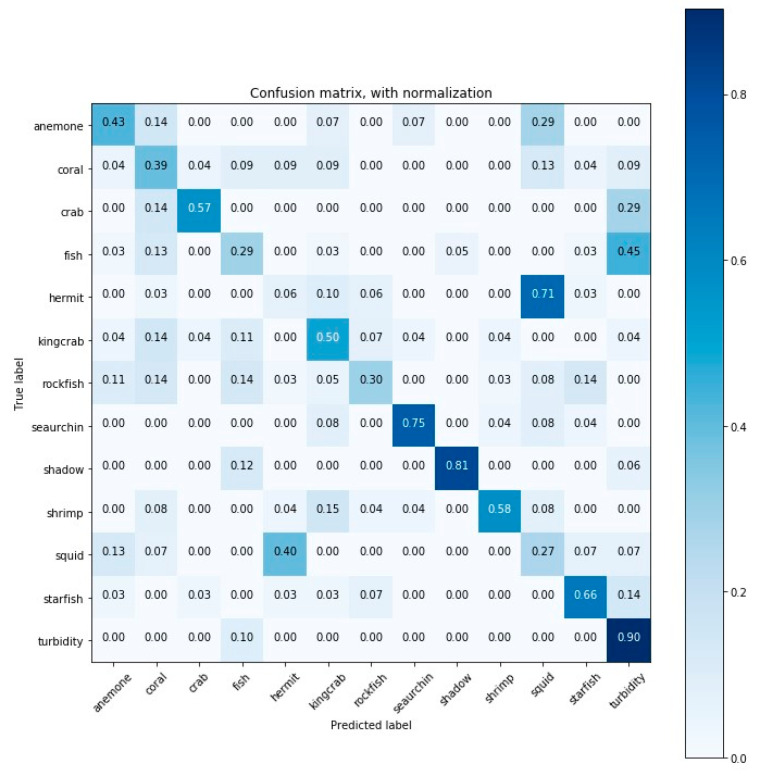
Confusion matrix for the classification results (accuracy) obtained by linear support vector machine and stochastic gradient descent (LSVM + SGD).

**Figure A3:**
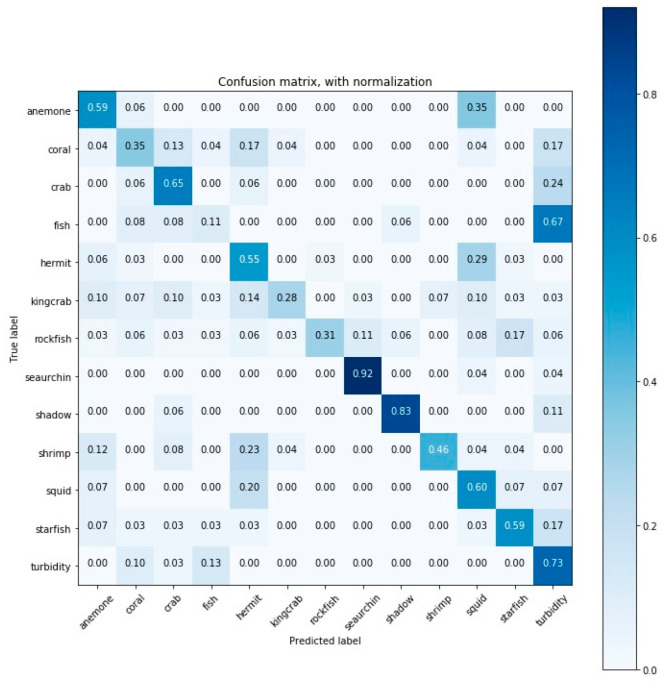
Confusion matrix for the classification results (accuracy) obtained by K-nearest neighbors (K-NN) (k = 39).

**Figure A4:**
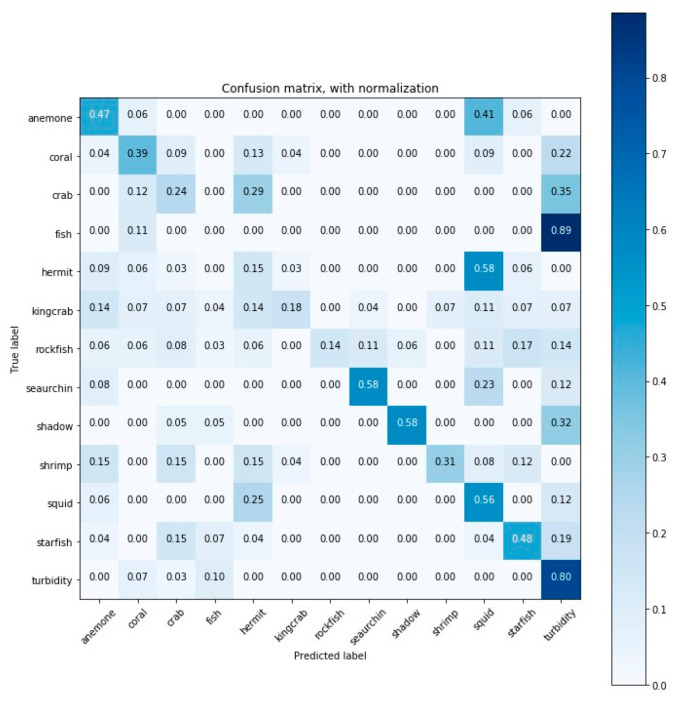
Confusion matrix for the classification results (accuracy) obtained by K-NN (k = 99).

**Figure A5:**
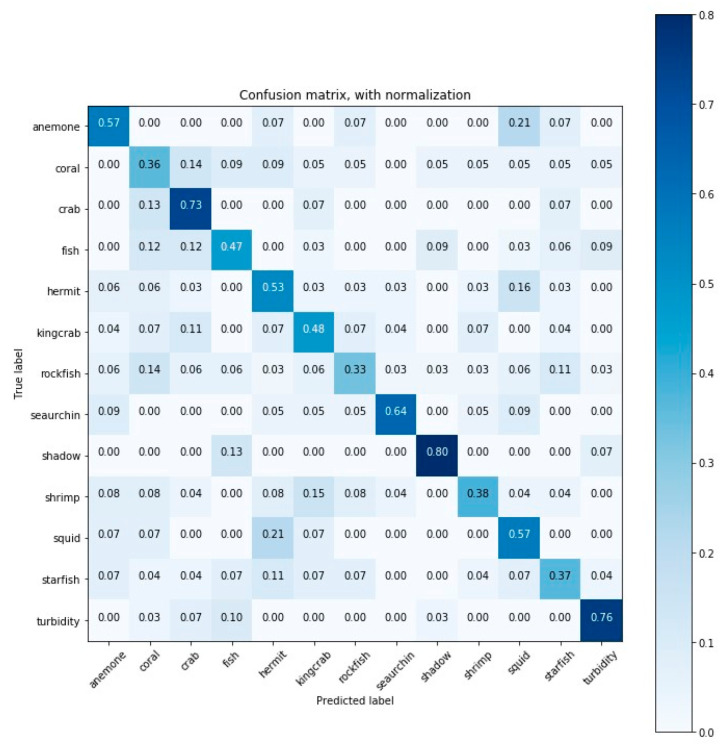
Confusion matrix for the classification results (accuracy) obtained by decision tree (DT) DT-1.

**Figure A6:**
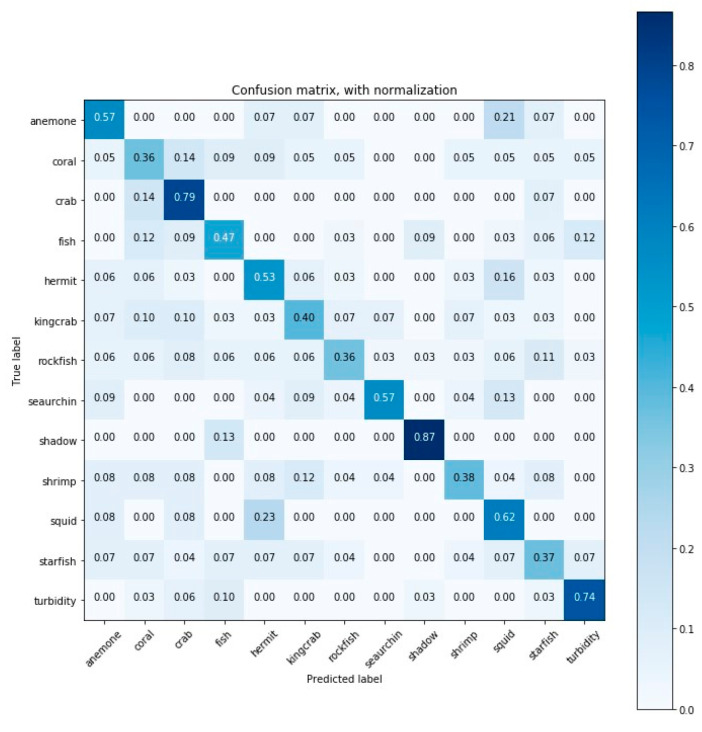
Confusion matrix for the classification results (accuracy) obtained by DT-2.

**Figure A7:**
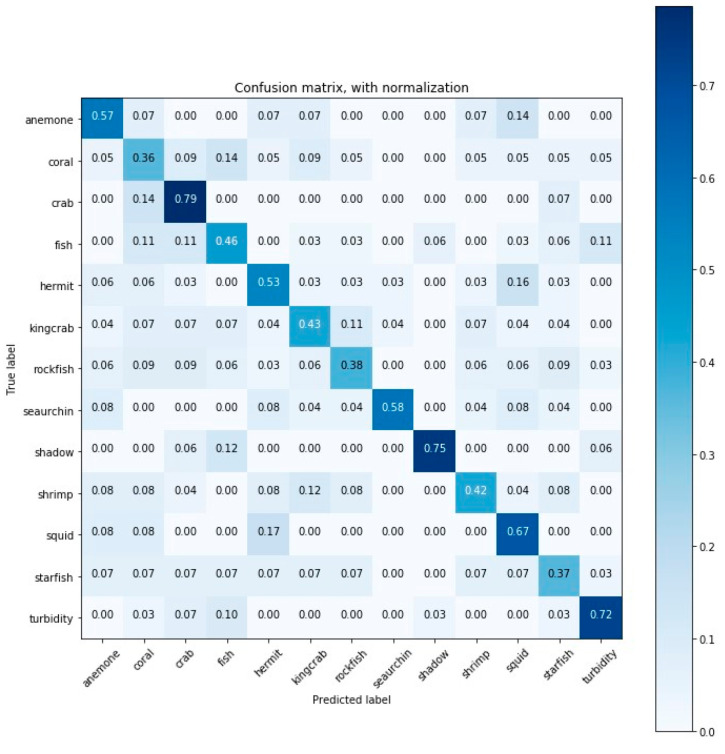
Confusion matrix for the classification results (accuracy) obtained by RF-1.

**Figure A8:**
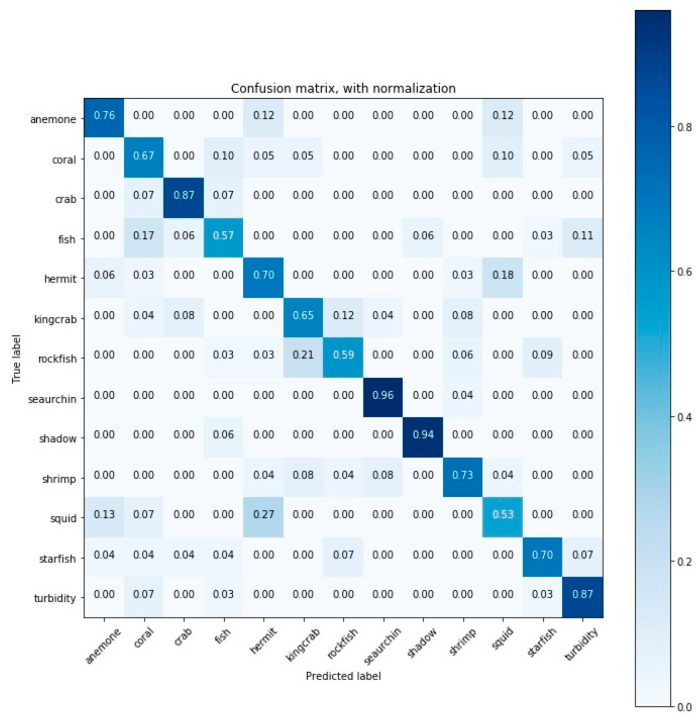
Confusion matrix for the classification results (accuracy) obtained by convolutional neural network (CNN) CNN-1.

**Figure A9:**
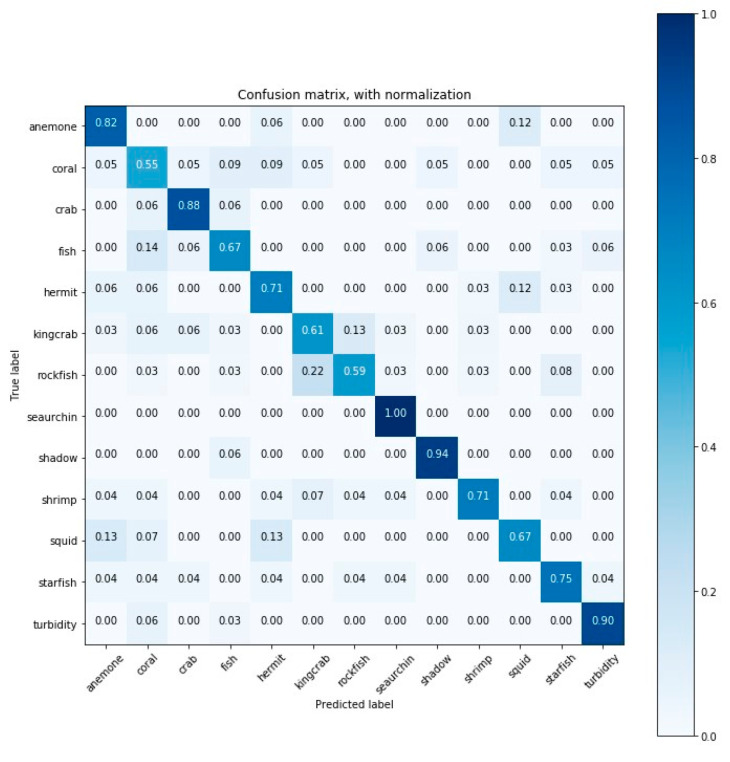
Confusion matrix for the classification results (accuracy) obtained by CNN-2.

**Figure A10:**
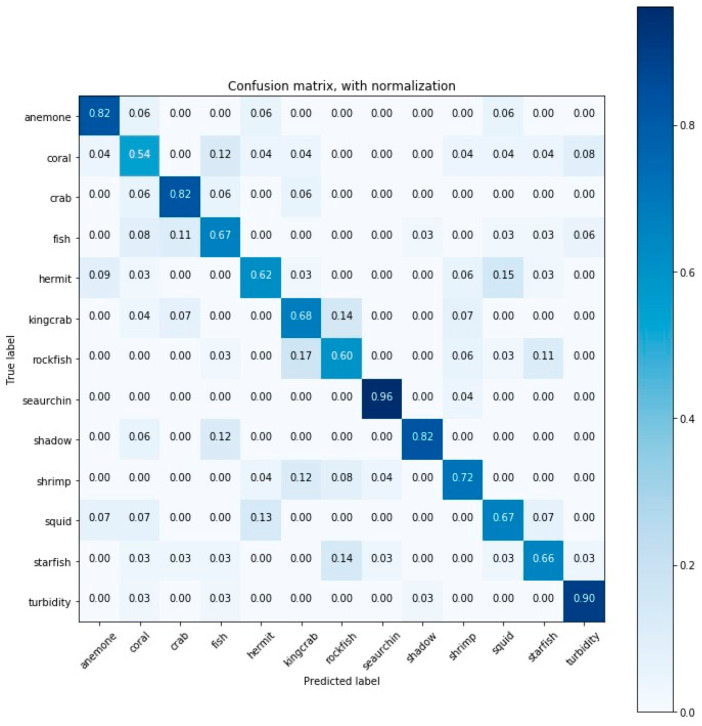
Confusion matrix for the classification results (accuracy) obtained by CNN-3.

**Figure A11:**
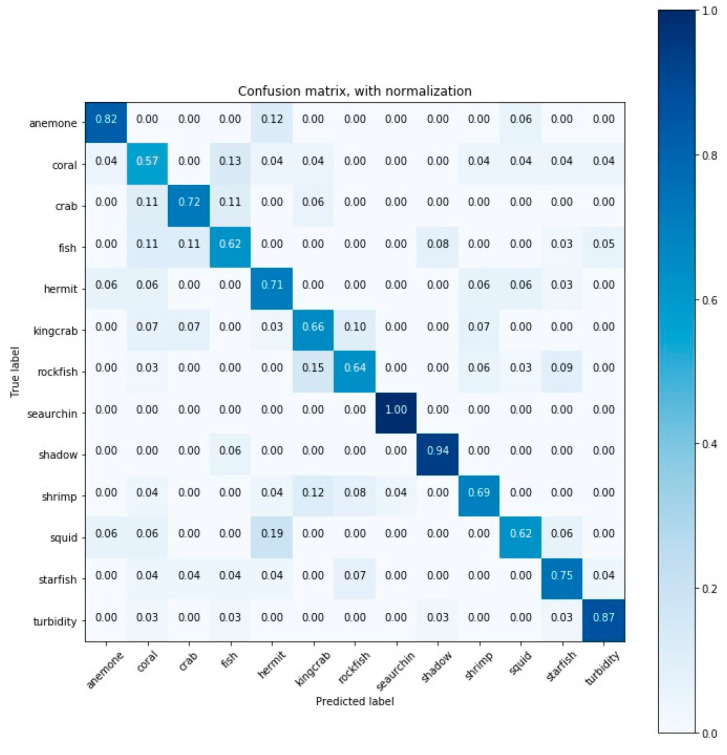
Confusion matrix for the classification results (accuracy) obtained by CNN-4.

**Figure A12:**
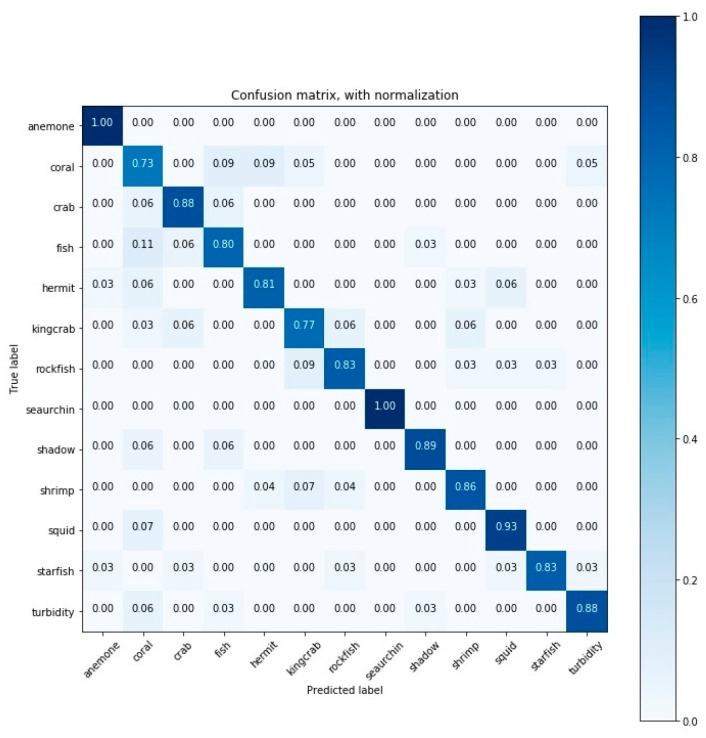
Confusion matrix for the classification results (accuracy) obtained by DNN-2.

**Figure A13:**
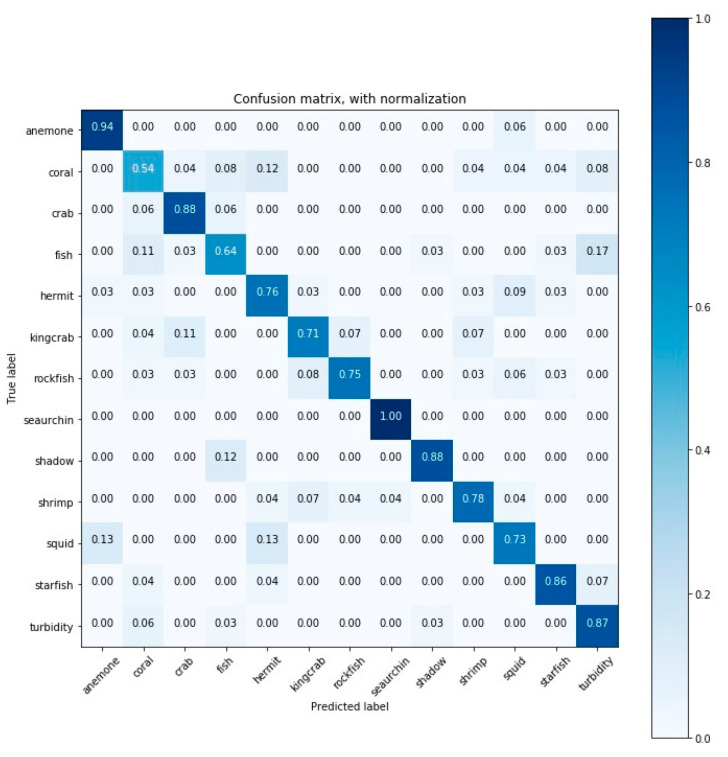
Confusion matrix for the classification results (accuracy) obtained by DNN-3.

**Figure A14:**
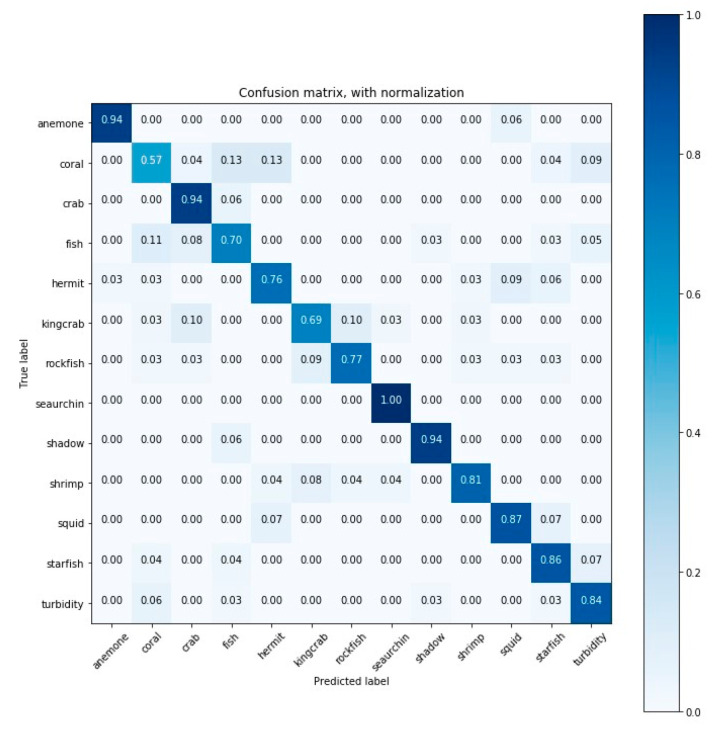
Confusion matrix for the classification results (accuracy) obtained by DNN-4.

## Appendix A

This appendix contains the rest of the confusion matrices of the results obtained on the test dataset from Table 4.
