# Correction: Lopez-Vazquez et al. Video Image Enhancement and Machine Learning Pipeline for Underwater Animal Detection and Classification at Cabled Observatories. *Sensors* 2020, *20*, 726

**DOI:** 10.3390/s23010016

**Published:** 2022-12-20

**Authors:** Vanesa Lopez-Vazquez, Jose Manuel Lopez-Guede, Simone Marini, Emanuela Fanelli, Espen Johnsen, Jacopo Aguzzi

**Affiliations:** 1 DS Labs, R+D+I unit of Deusto Sistemas S.A., 01015 Vitoria-Gasteiz, Spain; 2 University of the Basque Country (UPV/EHU), Nieves Cano, 12, 01006 Vitoria-Gasteiz, Spain; 3 Department of System Engineering and Automation Control, Faculty of Engineering of Vitoria-Gasteiz, University of the Basque Country (UPV/EHU), Nieves Cano, 12, 01006 Vitoria-Gasteiz, Spain; 4 Institute of Marine Sciences, National Research Council of Italy (CNR), 19032 La Spezia, Italy; 5 Stazione Zoologica Anton Dohrn (SZN), 80122 Naples, Italy; 6 Department of Life and Environmental Sciences, Polytechnic University of Marche, Via Brecce Bianche, 60131 Ancona, Italy; 7 Institute of Marine Research, P.O. Box 1870, 5817 Bergen, Norway; 8 Instituto de Ciencias del Mar (ICM) of the Consejo Superior de Investigaciones Científicas (CSIC), 08003 Barcelona, Spain

The authors wish to correct the following error in the original paper [1].

An additional affiliation (University of the Basque Country (UPV/EHU), Nieves Cano, 12, 01006, Vitoria-Gasteiz, Spain) has been added to the first author. Due to this change, the numbers of the rest of the affiliations of each author were updated.

The authors apologize for any inconvenience caused and state that the scientific conclusions are unaffected. This correction was approved by the academic editor. The original publication has also been updated.
